# Endoscopic submucosal dissection for a retrorectal tailgut cyst: a case report

**DOI:** 10.1055/a-2749-3464

**Published:** 2026-01-15

**Authors:** Jiawei Lin, Jing Wu, Min Lin

**Affiliations:** 1 599923 Department of Gastroenterology, Changzhou Second Peopleʼs Hospital of Nanjing Medical University, Changzhou, China; 2 Nanjing Medical University, Nangjing, China

A 61-year-old woman was referred to our clinic with a 2-year history of unprovoked lower abdominal pain, which was partially relieved by bending forward. Magnetic resonance imaging of the pelvis showed that T1-weighted images revealed isointense signal intensity, while T2-weighted images revealed high signal intensity, consistent with a multiloculated cystic lesion ( Fig. 1 ). Endoscopic ultrasound identified a 4 cm hypoechoic cystic lesion originating from the lamina propria ( Fig. 2 ).

**Fig. 1:**
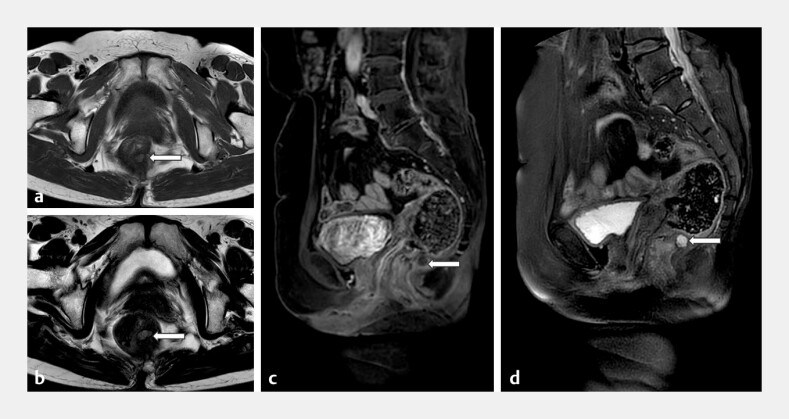
Preoperative MRI. **a** An axial T1-weighted MR image shows the well-defined, thin-walled cyst with isointense signal intensity (arrow). **b** An axial T2-weighted image shows high signal intensity (arrow). **c** A contrast-enhanced sagittal T1-weighted image shows a ring-like enhancement of its internal nodularity (arrow). **d** Sagittal fat-suppressed T2-weighted imaging (SPAIR) shows heterogeneous hyperintense signals, with nodular long T1 and long T2 signals noted internally (arrow). MRI, magnetic resonance imaging.

**Fig. 2:**
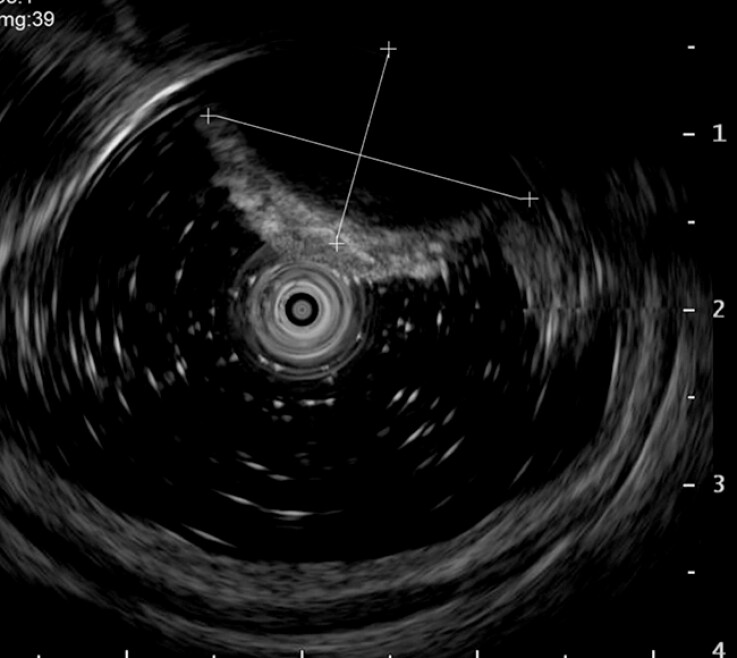
Transrectal endoscopic ultrasound showing a 4-cm hypoechoic mass.

The procedure was performed using endoscopic submucosal dissection (ESD; Video 1 ). The submucosa layer is the injected layer until the mucosa was sufficiently elevated. Oral mucosa incision was made with a Dual knife to expose the tumor, followed by dissection along the tumor margin with an IT knife until complete resection was achieved. After confirming the absence of active bleeding with a thermal coagulation forceps, purse-string suturing was performed using endoloop and metallic clips ( Fig. 3 **a–e** ). The resected specimen was finally retrieved using a snare ( Fig. 3 **f** ).

Endoscopic submucosal dissection for a retrorectal tailgut cyst.Video 1

**Fig. 3:**
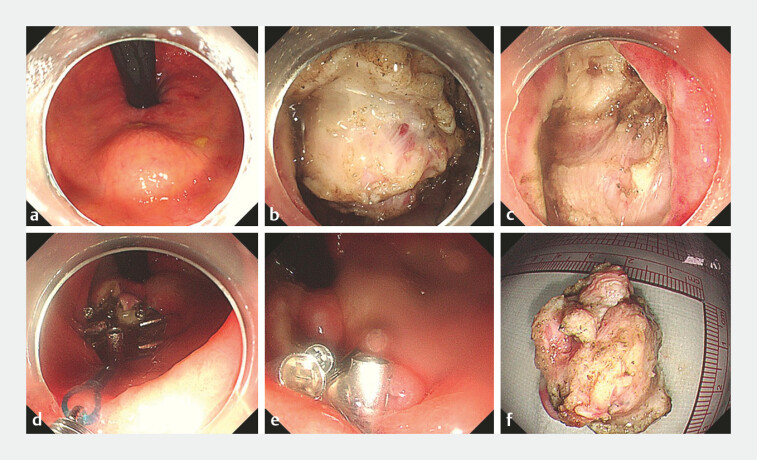
Endoscopic rectal mucosal dissection. **a** A hemispherical bulge with a diameter of about 4 cm in the posterior rectal wall. **b** Circumferential incision and dissection of the lesion. **c** The wound following submucosal dissection. **d, e** Metallic clips combined with endoloop for purse-string closure. **f** The resected mass for pathological evaluation.

The patient made a swift recovery and was discharged 8 days after the procedure. Follow-up computed tomography confirmed the complete removal of the cyst ( Fig. 4 ). The postoperative pathological report showed a cystic structure lined by pseudostratified ciliated columnar epithelium, surrounded by hyperplastic smooth muscle bundles and focal chronic inflammation ( Fig. 5 ). These features were diagnostic of a tailgut cyst, with no evidence of malignancy.

**Fig. 4:**
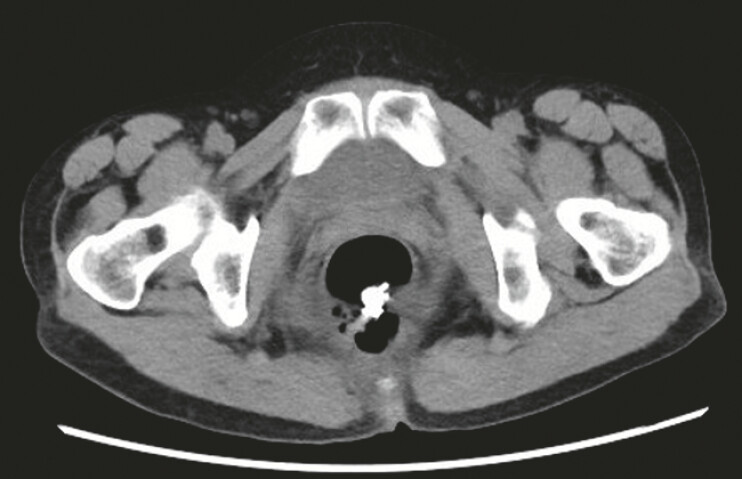
A postoperative CT scan confirming the complete excision of the cyst. CT, computed tomography.

**Fig. 5:**
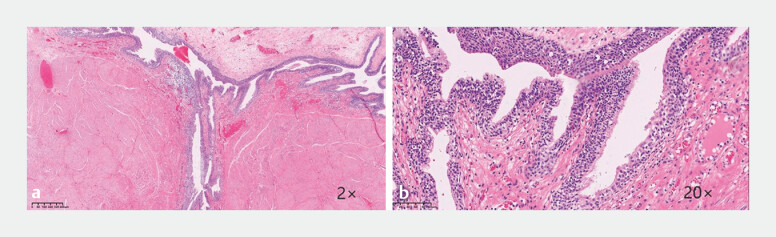
Histopathological findings. **a, b** Hematoxylin and eosin staining. The cystic lesion is lined by pseudostratified ciliated columnar epithelium. Smooth muscle tissue hyperplasia is visible around the cyst, with focal inflammation present in the cyst wall.

Accurate diagnosis and differentiation of tailgut cysts rely on detailed preoperative imaging and histopathological assessment. Given their potential for malignant transformation 1 , early intervention and appropriate treatment selection are crucial for patient prognosis. Complete surgical excision remains the standard treatment, as it effectively relieves symptoms and prevents complications including hemorrhage, infection, fistula formation, and malignancy 2 . As a natural orifice procedure, ESD avoids external incisions, which may result in reduced postoperative pain, faster recovery, and the absence of abdominal scarring. Thus, ESD represents a valuable addition to the therapeutic options for tailgut cysts.

Endoscopy_UCTN_Code_TTT_1AQ_2AD_3AD

## Notes

Endoscopy E-Videos https://eref.thieme.de/e-videos
*E-Videos* is an open access online section of the journal *Endoscopy* , reporting on interesting cases and new techniques in gastroenterological endoscopy. All papers include a high-quality video and are published with a Creative Commons CC-BY license. Endoscopy E-Videos qualify for HINARI discounts and waivers and eligibility is automatically checked during the submission process. We grant 100% waivers to articles whose corresponding authors are based in Group A countries and 50% waivers to those who are based in Group B countries as classified by Research4Life (see: https://www.research4life.org/access/eligibility/ ). This section has its own submission website at https://mc.manuscriptcentral.com/e-videos .
